# Effect of Hemoglobin A1c Trajectories on Future Outcomes in a 10-Year Cohort With Type 2 Diabetes Mellitus

**DOI:** 10.3389/fendo.2022.846823

**Published:** 2022-04-05

**Authors:** Chifa Ma, Weinan Zhang, Rongrong Xie, Gang Wan, Guangran Yang, Xuelian Zhang, Hanjing Fu, Liangxiang Zhu, Yujie Lv, Jiandong Zhang, Yuling Li, Yu Ji, Dayong Gao, Xueli Cui, Ziming Wang, Yingjun Chen, Shenyuan Yuan, Mingxia Yuan

**Affiliations:** ^1^ Department of Endocrinology, Beijing Friendship Hospital, Capital Medical University, Beijing, China; ^2^ Department of Endocrinology, Beijing Tongren Hospital, Capital Medical University, Beijing, China; ^3^ Medical Records and Statistics Department, Beijing Ditan Hospital, Capital Medical University, Beijing, China; ^4^ Department of General Practice, Cuigezhuang Community Health Service Center, Beijing, China; ^5^ Department of General Practice, Jinsong Community Health Service Center, Beijing, China; ^6^ Department of General Practice, Xinjiekou Community Health Service Center, Beijing, China; ^7^ Department of Endocrinology, Beijing Aerospace General Hospital, Beijing, China; ^8^ Department of General Practice, Aerospace Central Hospital, Beijing, China; ^9^ Department of General Practice, Sanlitun Community Health Service Center, Beijing, China; ^10^ Department of General Practice, Jiangtai Community Health Service Center, Beijing, China; ^11^ Department of General Practice, Majiapu Community Health Service Center, Beijing, China

**Keywords:** clinical outcomes, HbA1c-adjSD, HbA1c-CV, HbA1c trajectories, HbA1c variability, type 2 diabetes mellitus

## Abstract

**Background:**

Hemoglobin A1c (HbA1c) variability may be a predictor of diabetic complications, but the predictive values of HbA1c trajectories remain unclear. We aimed to classify long-term HbA1c trajectories and to explore their effects on future clinical outcomes in a 10-year cohort with type 2 diabetes mellitus (T2DM).

**Methods:**

A total of 2,161 participants with T2DM from the Beijing Community Diabetes Study were included. The 10-year follow-up was divided into two stages for the present data analysis. Stage I (from 2008 to 2014) was used to identify the HbA1c trajectories and to calculate the adjusted SD of HbA1c (HbA1c-adjSD), or the coefficient of variation of HbA1c (HbA1c-CV). Stage II (from 2014 to 2018) was used to collect the records of the new occurrence of diabetes-related clinical outcomes. Latent growth mixture models were used to identify HbA1c trajectories. Cox proportional hazards models were used to explore the relationship between HbA1c trajectories, HbA1c-adjSD, or HbA1c-CV and the future outcomes.

**Results:**

Three HbA1c trajectories were identified, including low stable (88.34%), gradual decreasing (5.83%), and pre-stable and post-increase (5.83%). Either the risk of death or the chronic complications were significantly higher in the latter two groups compared to the low stable group after adjustment for average HbA1c and other traditional risk factors, the adjusted hazard ratios (HRs) for renal events, composite endpoint, and all-cause death for the pre-stable and post-increase group were 2.83 [95%CI: 1.25–6.41, p = 0.013], 1.85 (95%CI: 1.10–3.10, p = 0.020), and 3.01 (95%CI: 1.13–8.07, p = 0.028), respectively, and the adjusted HR for renal events for the gradual decreasing group was 2.37 (95%CI: 1.08–5.21, p = 0.032). In addition, both univariate and multivariate Cox HR models indicated that participants in the fourth and third quartiles of HbA1c-CV or HbA1c-adjSD were at higher risk of renal events compared to participants in the first quartile.

**Conclusions:**

HbA1c trajectories, HbA1c-CV, and HbA1c-adjSD could all predict diabetes-related clinical outcomes. HbA1c trajectories could reflect long-term blood glucose fluctuation more intuitively, and non-stable HbA1c trajectories may predict increased risk of renal events, all-cause death, and composite endpoint events, independent of average HbA1c.

## Introduction

With the development of the economy and the change of lifestyle, the prevalence of diabetes increased significantly from 0.67% in China in 1980 to 11.2% in 2015–2017 (1, 2), and 76.4% of diabetic individuals suffered from over one kind of complications (3), which are the main causes of death in diabetes patients and contribute to significant economic burdens (4). Thus, it is an urgent issue to identify risk factors of diabetic complications and to implement timely interventions to prevent them.

Mounting evidence indicates that high average blood glucose level, usually estimated by hemoglobin A1c (HbA1c), is closely associated with diabetic complications and clinical outcomes (5, 6). However, normal or close-to-normal HbA1c with higher glucose fluctuation may be even more dangerous due to inducing pathogenic mechanisms related to the occurrence of diabetic complications (7). Glycemic control or HbA1c value is changing over the process of diabetes due to different factors contributing to the shape of HbA1c trajectories, such as the change of lifestyle, different treatment, aging, and other concomitant diseases, while most studies focused on the value of HbA1c at one time point and ignored the long-term fluctuation of blood glucose. HbA1c variability, one of the indicators that could reflect the long-term glucose fluctuation, including the SD of HbA1c (HbA1c-SD) and the coefficient of variation of HbA1c (HbA1c-CV), may be associated with diabetic complications (8), but these simple indicators could not intuitively reflect the complicated fact of glucose varying with time, while HbA1c trajectories could make up this gap to some extent, but the clinical value of HbA1c trajectories did not get deserved concern. There were several HbA1c trajectories that have been identified in a few studies that may be associated with diabetes complications (9–11). However, related studies on the relationships between HbA1c trajectories and future clinical outcomes are limited, let alone the studies based on the Chinese diabetic population. Therefore, we aimed to identify the trajectories of long-term HbA1c values in a Chinese population with type 2 diabetes mellitus (T2DM) and to confirm the role of HbA1c trajectories on future diabetes-related clinical outcomes.

## Materials and Methods

### Study Population

Participants with T2DM from 14 communities in Beijing were recruited from Beijing Community Diabetes Study between August 2008 and July 2009, which has been described in detail previously (12). The participants enrolled in the study were followed up for 10 years (2008–2018). On account of most of the clinical outcomes (76%) that occurred after 2014, we divided the 10-year follow-up into two stages for the present data analysis to explore the effect of HbA1c trajectories on future clinical outcomes. Stage I (from 2008 to 2014) was used to identify the HbA1c trajectories and to calculate the HbA1c-SD and HbA1c-CV. Stage II (from 2014 to 2018) was used to collect the records of the occurrence of future diabetes-related clinical outcomes.

**Figure 1:**
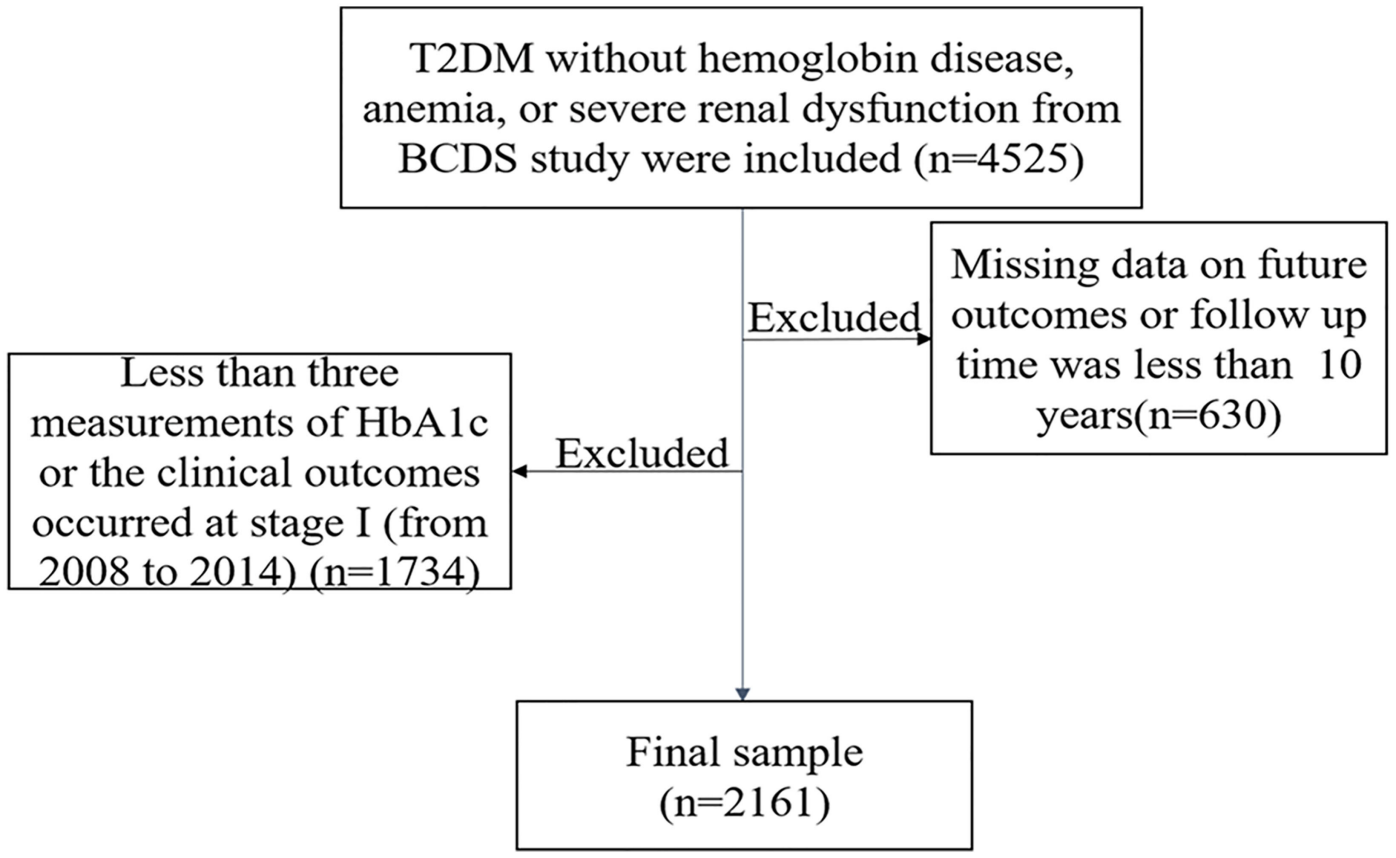
The flowchart of the study. T2DM, type 2 diabetes mellitus; BCDS, Beijing Community Diabetes Study.

T2DM was defined according to the criteria of the WHO (1999) (13). A total of 4,525 T2DM participants without hemoglobin disease, anemia, or severe renal dysfunction were included in the initial study, while those with missing data of outcomes or who did not finish the 10-year follow-up were excluded (n = 630). Participants who had less than three measurements of HbA1c or with the clinical outcomes that occurred at stage I (from 2008 to 2014) were also excluded (n = 1,734). A final total of 2,161 participants were included (**Figure 1**).

All the T2DM patients were managed by the community doctors and specialists from Beijing Tongren Hospital. Patients were given health education, including individualized guidance on reasonable diet and exercise. During the annual follow-up, questionnaire survey, physical examination, and the biochemical indexes evaluation were conducted, and electrocardiogram and eye examination were also performed simultaneously.

### Data Collection

The date of birth, gender, duration of diabetes, concomitant diseases, smoking habits, income, education, and medical history at baseline were collected using a questionnaire by trained interviewers. The income was divided into three categories: less than 2,000, 2,000 to 4,000, and over 4,000 yuan per month. Participant education was categorized into three groups, namely, primary education (illiterate and primary school graduates), secondary education (junior and senior high school graduates), and higher education (college graduates and above). Anti-hyperglycemic therapy was classified as non-drug therapy, oral anti-hyperglycemic drugs, insulin treatment, and oral anti-hyperglycemic drugs combined with insulin. Blood pressure, body mass index (BMI), and waist circumference (WC) were also collected using standard methods. Fasting blood samples were collected for measurements of HbA1c, blood glucose, serum creatinine, serum uric acid, alanine aminotransferase, and lipids. HbA1c was measured using a Bio-Rad Variant hemoglobin analyzer by a central endocrinology laboratory in Beijing Tongren Hospital.

### Outcomes

Outcomes of interest included all-cause death, cardiovascular events (acute myocardial infarction, coronary artery bypass grafting, coronary stent, unstable angina pectoris, hospitalization for heart failure, and installation of cardiac pacemaker), cerebrovascular events (cerebral hemorrhage, cerebral infarction, transient ischemic attack, and subarachnoid hemorrhage), renal events (new-onset proteinuria, microalbuminuria turning into macroalbuminuria, doubling of the serum creatinine level, and dialysis), diabetic eye disease events (fundus laser and vitrectomy treatment), and composite endpoint events (all-cause death, cardiovascular events, cerebrovascular events, renal events, and diabetic eye disease events), which all occurred from 2014 to 2018. All outcomes were adjudicated by an independent committee, which was in charge of assignments on validation of data and events based on the outcome criteria.

### Calculation of HbA1c-SD and HbA1c-CV

The HbA1c-SD and HbA1c-CV were calculated using HbA1c values measured in Stage I to evaluate HbA1c variability. As the number of HbA1c measurements would affect the SD value, the HbA1c-SD was adjusted for the number of HbA1c assessments according to the formula: adjusted SD of HbA1c $(\text{HbA}1\text{c}-\text{adjSD})=\text{HbA}1\text{c}-\text{SD/}\sqrt{n/(n-1)}$ (14). Furthermore, the HbA1c-CV was calculated as the HbA1c-SD to HbA1c–MEAN ratio to correct for larger SDs resulting from higher absolute HbA1c–MEAN values (14).

### Statistical Analysis

HbA1c trajectories were identified using the latent growth mixture model (LGMM) with Mplus 8.0 (11). We assumed that there were multiple potential growth trajectories in the population, and each potential trajectory represented a subclass that had different growth patterns. The model contains two kinds of latent variables, including continuous latent variables and classified latent variables. The former contains intercept and slope of growth characteristic parameters, while the latter refers to the mutually exclusive subgroups. We used potential subgroups in increasing order to fit linear and quadratic growth mixed models. The optimal model was selected through the information indexes, including the Lo–Mendell–Rubin fit index, bootstrapped likelihood ratio test, entropy, and the adjusted Bayesian information criterion. Each trajectory subgroup must be large enough to have clinical relevance, namely, containing a minimum of 1% of patients (11), and then we assigned patients to the subgroup where they had the highest posterior probability.

Normally distributed quantitative variables were expressed as the mean ± SD, whereas categorical variables were shown as numbers (percentages). Parameters that were not normally distributed were presented as the median [interquartile range (IQR)]. Comparison of variables among groups was performed using the Kruskal–Wallis and one-way ANOVA. Cox proportional hazards models were used to evaluate the relationship between HbA1c trajectories, HbA1c-CV, or HbA1c-adjSD, and future outcomes. SPSS (version 24.0) was used to perform all statistical analyses. p < 0.05 (two-sided) indicated statistical significance.

## Results

### Three Patterns of Hemoglobin A1c Trajectories and Their Baseline Characteristics

**Figure 2:**
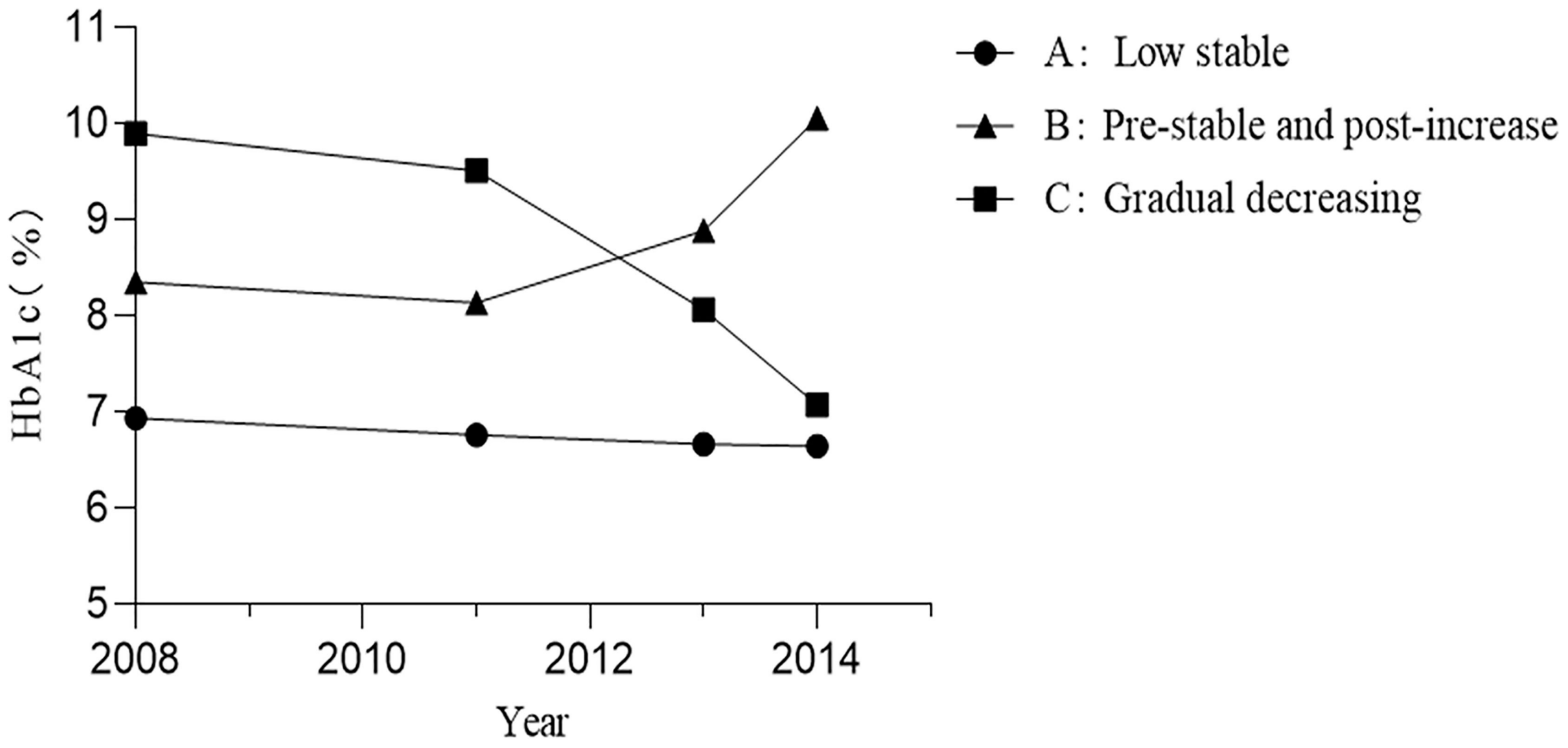
Three patterns of HbA1c trajectories. HbA1c, hemoglobin A1c.

As shown in **Figure 2**, three patterns of HbA1c trajectories were identified by LGMM, including the low stable (88.34%), pre-stable and post-increase (5.83%), and gradual decreasing (5.83%) HbA1c trajectories. The low stable group had a relatively good glycemic control with the lowest levels of HbA1c. The pre-stable and post-increase group had higher than recommended but stable HbA1c initially and significantly increased at the later stage, while those in the gradual decreasing group had the highest levels of HbA1c in the first few years but gradually decreased over time.

Baseline characteristics of three patterns of HbA1c trajectories groups are shown in **Table 1**. The median baseline fasting plasma glucose (FPG) of individuals was 6.8 (IQR, 6.0–8.0), 8.4 (IQR, 7.0-10.9), and 11.2 mmol/L (IQR, 9.0-13.6), and the median baseline HbA1c was 6.7% (IQR, 6.2–7.4), 8.0% (IQR, 7.2-9.7), and 9.9% (IQR, 9.1-11.5) in the low stable, pre-stable and post-increase, and gradual decreasing groups, respectively. Although the glycemic control was significantly different among the three groups, there were no significant differences in gender, T2DM duration, anti-hyperglycemic therapy, high-density lipoprotein cholesterol (HDL-C), alanine aminotransferase, systolic blood pressure (SBP), smoking habits, and the prevalence of hypertension, stroke, and dyslipidemia at baseline. Compared with participants in the low stable group, those in the pre-stable and post-increase and gradual decreasing groups were more likely to be younger and had higher BMI, diastolic blood pressure (DBP), WC, triglycerides (TG), total cholesterol (TC), and low-density lipoprotein cholesterol (LDL-C) but had lower income and proportion of higher education. We also found that participants in the pre-stable and post-increase group had lower levels of uric acid, a lower proportion of angiotensin-converting enzyme inhibitors (ACEIs) or angiotensin II receptor blockers (ARBs) takers, and a lower prevalence of coronary heart disease at baseline as compared to those in the low stable group. In addition, participants in the pre-stable and post-increase group also had the lowest levels of serum creatinine among the three groups.

**Table 1:** Baseline characteristics of three patterns of HbA1c trajectories.

	Low stable (n = 1,909)	Pre-stable and post-increase (n = 126)	Gradual decreasing (n = 126)	p
Age (year)	64 (56–71)	58 (50–68)^*^	59 (54–70)^*^	<0.001
Male, n (%)	737 (38.6)	51 (40.5)	43 (34.1)	0.540
Diabetes duration (year)	4.8 (1.4–10.1)	5.3 (0.0–11.2)	7.2 (1.9–12.3)	0.414
Income (yuan)				0.004
<2,000, n (%)	1004 (56.5)	83 (68.0)^*^	87 (70.7)^*^	
2,000~4,000, n (%)	673 (37.9)	35 (28.7)	30 (24.4)^*^	
≥4,000, n (%)	100 (5.6)	4 (3.3)	6 (4.9)	
Education				0.001
Primary, n (%)	298 (15.7)	22 (17.5)	30 (23.8)	
Secondary, n (%)	1197 (63.1)	90 (71.4)	84 (66.7)	
Higher, n (%)	403 (21.2)	14 (11.1)^*^	12 (9.5)^*^	
Concomitant disease, n (%)				
Hypertension	1316 (68.9)	75 (59.5)	82 (65.1)	0.067
Stroke	219 (11.5)	11 (8.7)	11 (8.7)	0.430
Coronary heart disease	390 (20.4)	10 (7.9)^*^	15 (11.9)	<0.001
Dyslipidemia	801 (42.0)	44 (34.9)	52 (41.3)	0.299
Anti-hyperglycemic therapy, n (%)				0.213
None	199 (10.4)	19 (15.1)	12 (9.5)	
Oral drug	1313 (68.8)	78 (61.9)	81 (64.3)	
Insulin	119 (6.2)	8 (6.3)	14 (11.1)	
Combined medication	278 (14.6)	21 (16.7)	19 (15.1)	
ACEI/ARB treatment, n (%)	537 (28.1)	17 (13.5)^*^	32 (25.4)	0.001
Smoking, n (%)	244 (12.8)	25 (19.8)	16 (12.7)	0.075
BMI (kg/m^2^)	25.09 ± 3.25	25.87 ± 3.43^*^	26.42 ± 3.29^*^	<0.001
WC (cm)	88.11 ± 9.20	89.91 ± 8.92	91.15 ± 9.25^*^	<0.001
SBP (mmHg)	128.66 ± 12.90	130.88 ± 16.14	129.44 ± 12.87	0.159
DBP (mmHg)	77.07 ± 8.16	80.29 ± 9.02^*^	79.27 ± 9.06^*^	<0.001
FPG (mmol/L)	6.8 (6.0–8.0)	8.4(7.0-10.9)*	11.2(9.0-13.6)*^#^	<0.001
HbA1c (%)	6.7 (6.2–7.4)	8.0(7.2-9.7)*	9.9(9.1-11.5)^*#^	<0.001
TC (mmol/L)	5.10 ± 1.19	5.55 ± 1.27^*^	5.36 ± 1.25	<0.001
TG (mmol/L)	1.5 (1.0–2.0)	1.7 (1.2–2.5)^*^	1.7 (1.2–2.4)^*^	<0.001
LDL-C (mmol/L)	2.99 ± 0.87	3.21 ± 0.99^*^	3.15 ± 0.93	0.005
HDL-C (mmol/L)	1.33 ± 0.44	1.25 ± 0.31	1.29 ± 0.39	0.104
ALT (U/L)	19.3 (14.0–26.8)	20.0 (14.6–30.0)	19.0 (13.2–28.5)	0.403
Uric acid (μmol/L)	296.82 ± 82.86	256.84 ± 80.74^*^	280.28 ± 90.69	<0.001
Cr (μmol/L)	73.77 ± 23.64	65.06 ± 16.82^*^	74.49 ± 25.10^#^	0.001

Data are presented as mean ± SD, number (percentage), or median [interquartile range (IQR)].

ACEI, angiotensin-converting enzyme inhibitors; ARB, angiotensin II receptor blockers; BMI, body mass index; WC, waist circumference; SBP, systolic blood pressure; DBP, diastolic blood pressure; FPG, fasting plasma glucose; HbA1c, hemoglobin A1c; TG, triglycerides; TC, total cholesterol; LDL-C, low-density lipoprotein cholesterol; HDL-C, high-density lipoprotein cholesterol; ALT, alanine aminotransferase; UA, uric acid; Cr, creatinine.

^*^Compared with the low stable group, a two-sided p < 0.05 was considered statistically significant.

^#^Compared with the pre-stable and post-increase group, a two-sided p < 0.05 was considered statistically significant.

### Cumulative Incidence of Diabetes-Related Clinical Outcomes in Type 2 Diabetes Mellitus Patients From 2014 to 2018

**Figure 3:**
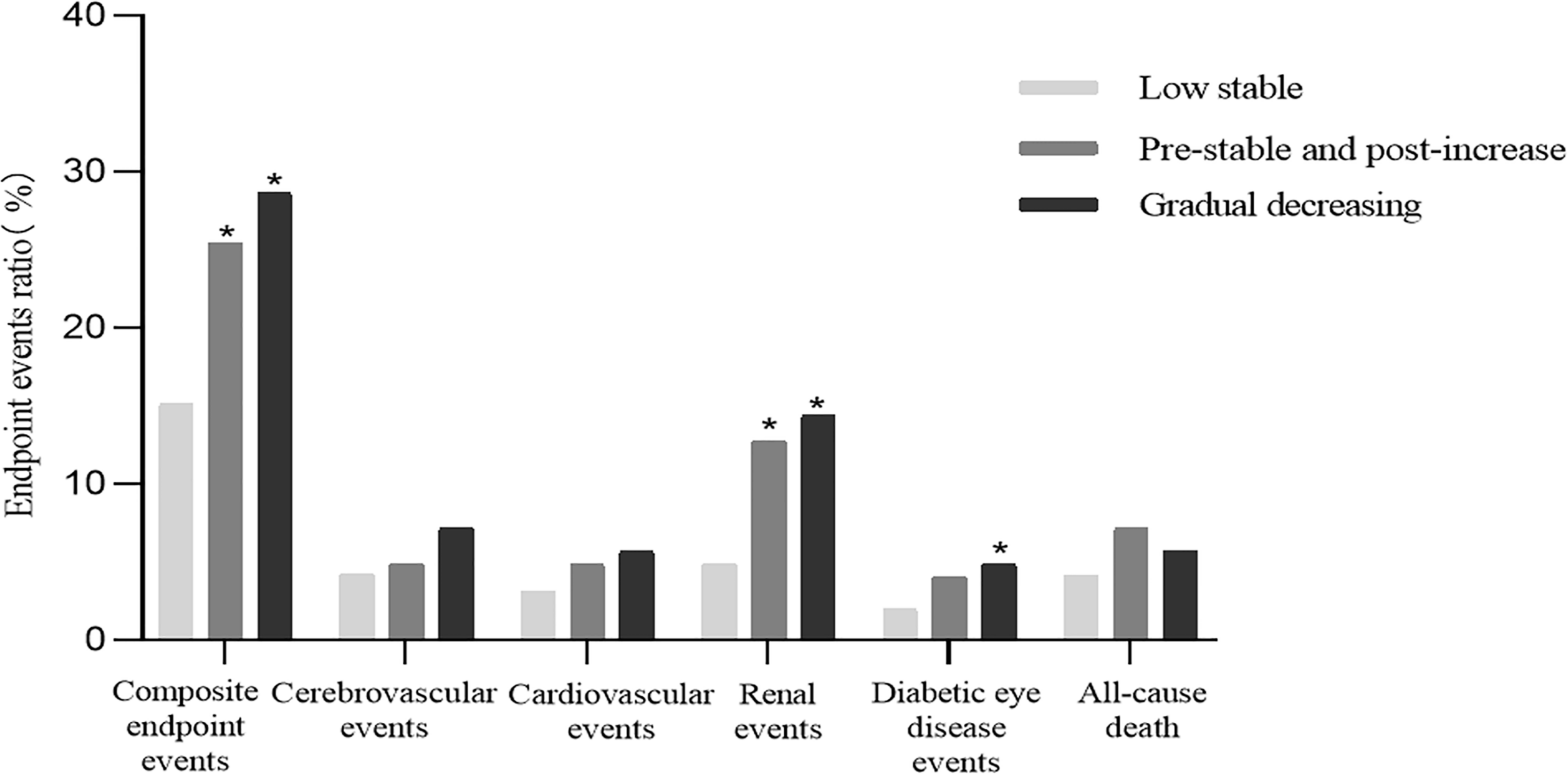
Cumulative incidence of diabetes-related clinical outcomes in T2DM patients from 2014 to 2018. ^*^Compared with the low stable group, a two-sided p < 0.05 was considered statistically significant. T2DM, type 2 diabetes mellitus.

From 2014 to 2018, 96 cardiovascular events, 70 cerebrovascular events, 125 renal events, 47 diabetic eye disease events, 356 composite endpoint events, and 94 cases of all-cause death occurred among 2,161 individuals with T2DM. As shown in **Figure 3**, compared to the low stable group, the incidence of renal events and composite endpoint events was significantly higher in the pre-stable and post-increase and gradual decreasing groups (both p < 0.001), and the prevalence of diabetic eye disease events was also significantly higher in the latter group (p < 0.05).

### Cox Proportional Hazards Models of the Hemoglobin A1c Trajectories With Future Diabetes-Related Clinical Outcomes

Cox proportional hazards models of the HbA1c trajectories with future clinical outcomes were shown in **Table 2**. In unadjusted analyses (Model 1), both the pre-stable and post-increase and gradual decreasing groups had a higher risk of renal events than the low stable group (both p < 0.001). After adjustment for gender, age, duration of T2DM, BMI, smoking habits, baseline concomitant diseases, TG, LDL-C, blood pressure, anti-hyperglycemic therapy, and ACEI or ARB treatment (Model 2), the increased risk of renal events remained statistically significant for the pre-stable and post-increase [hazard ratio (HR) = 2.91, 95%CI: 1.60–5.31, p < 0.001] and gradual decreasing (HR = 2.44, 95%CI: 1.37–4.34, p = 0.002) groups, respectively. In further adjustment for average HbA1c, the main trend of the risk of renal events for the pre-stable and post-increase (HR = 2.83, 95%CI: 1.25–6.41, p = 0.013) and gradual decreasing (HR = 2.37, 95%CI: 1.08–5.21, p = 0.032) groups remained significant (Model 3). The gradual decreasing group also had a higher risk of diabetic eye disease events (HR = 2.62, 95%CI: 1.10–6.22, p = 0.029) and composite endpoint events (HR = 2.00, 95%CI: 1.41–2.83, p < 0.001) than the low stable group in unadjusted analyses (Model 1). However, after adjustment for gender, age, duration of T2DM, BMI, smoking habits, baseline concomitant diseases, TG, LDL-C, blood pressure, anti-hyperglycemic therapy, ACEI or ARB treatment, and average HbA1c (Model 3), these differences were no longer significant (both p > 0.05). For macrovascular events, the association between cardiovascular events or cerebrovascular events and HbA1c trajectories did not reach a significant difference in the models without and with adjustments (all p > 0.05, Models 1 to 3). In addition, the pre-stable and post-increase group had a higher risk of all-cause death (HR = 3.02, 95%CI: 1.46–6.24 p = 0.003) and composite endpoint events (HR = 2.12, 95%CI: 1.42–3.15, p < 0.001) than the low stable group after adjustment for gender, age, duration of T2DM, BMI, smoking habits, baseline concomitant diseases, TG, LDL-C, blood pressure, anti-hyperglycemic therapy, and ACEI or ARB treatment (Model 2), and these associations remained statistically significant after additional adjustment for average HbA1c (both p < 0.05, Model 3).

**Table 2:** Cox proportional hazards models of the HbA1c trajectories with future diabetes-related clinical outcomes.

Clinical outcomes	Groups	Model 1		Model 2		Model 3	
		HR (95%CI)	p	HR (95%CI)	p	HR (95%CI)	p
Renal events			<0.001		<0.001		0.031
	A	Reference		Reference		Reference	
	B	3.05 (1.79, 5.19)	<0.001	2.91 (1.60, 5.31)	<0.001	2.83 (1.25, 6.41)	0.013
	C	3.13 (1.89, 5.20)	<0.001	2.44 (1.37, 4.34)	0.002	2.37 (1.08, 5.21)	0.032
Diabetic eye disease events			0.032		0.682		0.892
	A	Reference		Reference		Reference	
	B	2.30 (0.90, 5.86)	0.081	1.61 (0.55, 4.73)	0.389	0.96 (0.23, 3.98)	0.957
	C	2.62 (1.10, 6.22)	0.029	1.18 (0.36, 3.90)	0.790	0.71 (0.15, 3.30)	0.659
Cardiovascular events			0.236		0.171		0.708
	A	Reference		Reference		Reference	
	B	1.25 (0.55, 2.87)	0.593	1.86 (0.79, 4.41)	0.158	1.52 (0.52, 4.48)	0.449
	C	1.79 (0.90, 3.56)	0.098	1.76 (0.80, 3.88)	0.161	1.42 (0.50, 4.08)	0.511
Cerebrovascular events			0.115		0.287		0.977
	A	Reference		Reference		Reference	
	B	1.78 (0.77, 4.12)	0.181	1.83 (0.71, 4.72)	0.214	1.08 (0.33, 3.47)	0.902
	C	1.99 (0.91, 4.37)	0.085	1.69 (0.69, 4.14)	0.255	0.94 (0.29, 3.12)	0.923
All-cause death			0.120		0.005		0.087
	A	Reference		Reference		Reference	
	B	1.96 (0.98, 3.90)	0.056	3.02 (1.46, 6.24)	0.003	3.01 (1.13, 8.07)	0.028
	C	1.45 (0.67, 3.15)	0.344	2.03 (0.92, 4.48)	0.079	2.03 (0.72, 5.69)	0.178
Composite endpoint events			<0.001		<0.001		0.057
	A	Reference		Reference		Reference	
	B	1.89 (1.31, 2.73)	0.001	2.12 (1.42, 3.15)	<0.001	1.85 (1.10, 3.10)	0.020
	C	2.00 (1.41, 2.83)	<0.001	1.77 (1.20, 2.61)	0.004	1.54 (0.92, 2.59)	0.100

Group A (low stable) was regarded as the reference category. Group B: pre-stable and post-increase. Group C: gradual decreasing. Model 1 was a univariate analysis. Model 2 was adjusted for gender, age, duration of type 2 diabetes, body mass index, smoking habits, baseline concomitant disease, triglycerides; low-density lipoprotein cholesterol, blood pressure, anti-hyperglycemic therapy, and angiotensin-converting enzyme inhibitor or angiotensin II receptor blocker treatment. Model 3 was adjusted for the covariates of Model 2 plus average HbA1c.

HbA1c, hemoglobin A1c; HR, hazard ratio.

### Association of the HbA1c-adjSD and HbA1c-CV With Future Diabetes-Related Clinical Outcomes

We also explored the association of HbA1c-adjSD and HbA1c-CV quartiles with future clinical outcomes (**Tables 3**, **4**). In unadjusted analysis (Model 1), using quartile1 of the HbA1c-adjSD as a reference, the HRs for renal events were 1.28 (95%CI: 0.68–2.40, p = 0.443), 2.28 (95%CI: 1.30–4.00, p = 0.004), and 2.89 (95%CI: 1.68–4.96, p < 0.001) for quartile2, quartile3, and quartile4, respectively, and p for trend<0.001 (**Table 3**). After adjustment for gender, age, duration of T2DM, BMI, smoking habits, baseline concomitant diseases, TG, LDL-C, blood pressure, anti-hyperglycemic therapy, and ACEI or ARB treatment, this trend remained significant (p for trend = 0.001, Model 2). In further adjustment for average HbA1c, the HRs were 1.32 (95%CI: 0.67–2.59, p = 0.417), 2.40 (95%CI: 1.29–4.46, p = 0.006), and 2.40 (95%CI: 1.23–4.66, p = 0.010) for quartile2, quartile3, and quartile4, respectively, and p for trend = 0.014 (Model 3). We also found that the risk of composite endpoint events was significantly higher in the third quartile than the first quartile of the HbA1c-adjSD after adjustment for average HbA1c and other variables (HR = 1.53, 95%CI: 1.09–2.15, p = 0.014, Model 3, **Table 3**). Similarly, both univariate and multivariate Cox HR models (all p for trend <0.05, Models 1–3) indicated that participants in the fourth and third quartiles of the HbA1c-CV were at higher risk of renal events compared to participants in the first quartile (**Table 4**). The risk of composite endpoint events was significantly higher in the third and fourth quartiles than in the first quartile of the HbA1c-CV after adjustment for gender, age, duration of T2DM, BMI, smoking habits, baseline concomitant diseases, TG, LDL-C, blood pressure, anti-hyperglycemic therapy, and ACEI or ARB treatment (p for trend = 0.012, Model 2); however, in further adjustment for average HbA1c, these associations were no longer significant (p for trend = 0.164, Model 3, **Table 4**).

**Table 3:** Cox proportional hazards models of the HbA1c-adjSD with future diabetes-related clinical outcomes.

Clinical outcomes	HbA1c-adjSD	Model 1		Model 2		Model 3	
		HR (95%CI)	p	HR (95%CI)	p	HR (95%CI)	p
Renal events							
	Quartile1	Reference		Reference		Reference	
	Quartile2	1.28 (0.68–2.40)	0.443	1.34 (0.68–2.63)	0.393	1.32 (0.67–2.59)	0.417
	Quartile3	2.28 (1.30–4.00)	0.004	2.51 (1.36–4.65)	0.003	2.40 (1.29–4.46)	0.006
	Quartile4	2.89 (1.68–4.96)	<0.001	2.86 (1.58–5.18)	0.001	2.40 (1.23–4.66)	0.010
		p for trend	<0.001	p for trend	0.001	p for trend	0.014
Diabetic eye disease events							
	Quartile1	Reference		Reference		Reference	
	Quartile2	0.72 (0.30–1.77)	0.476	0.77 (0.29–2.03)	0.594	0.76 (0.29–2.01)	0.580
	Quartile3	0.72 (0.29–1.76)	0.471	0.69 (0.26–1.84)	0.460	0.67 (0.25–1.80)	0.429
	Quartile4	1.65 (0.80–3.40)	0.175	1.47 (0.66–3.31)	0.348	1.31 (0.50–3.41)	0.584
		p for trend	0.108	p for trend	0.306	p for trend	0.542
Cardiovascular events							
	Quartile1	Reference		Reference		Reference	
	Quartile2	1.20 (0.68–2.10)	0.526	1.14 (0.61–2.15)	0.683	1.12 (0.59–2.11)	0.733
	Quartile3	0.96 (0.53–1.74)	0.902	0.88 (0.44–1.74)	0.713	0.80 (0.40–1.60)	0.531
	Quartile4	1.15 (0.66–2.02)	0.616	1.13 (0.60–2.14)	0.708	0.80 (0.38–1.69)	0.558
		p for trend	0.847	p for trend	0.853	p for trend	0.735
Cerebrovascular events							
	Quartile1	Reference		Reference		Reference	
	Quartile2	0.75 (0.34–1.70)	0.494	0.77 (0.31–1.92)	0.575	0.75 (0.30–1.89)	0.546
	Quartile3	1.86 (0.97–3.58)	0.063	1.69 (0.80–3.58)	0.171	1.57 (0.74–3.34)	0.245
	Quartile4	1.52 (0.77–2.98)	0.228	1.67 (0.79–3.56)	0.183	1.22 (0.51–2.90)	0.654
		p for trend	0.057	p for trend	0.159	p for trend	0.340
All-cause death							
	Quartile1	Reference		Reference		Reference	
	Quartile2	0.91 (0.49–1.71)	0.769	0.70 (0.33–1.45)	0.332	0.68 (0.33–1.42)	0.306
	Quartile3	1.61 (0.93–2.78)	0.092	1.86 (1.02–3.38)	0.042	1.72 (0.94–3.15)	0.080
	Quartile4	1.12 (0.62–2.02)	0.718	1.38 (0.73–2.61)	0.315	1.01 (0.48–2.12)	0.978
		p for trend	0.181	p for trend	0.024	p for trend	0.037
Composite endpoint events							
	Quartile1	Reference		Reference		Reference	
	Quartile2	1.14 (0.82–1.58)	0.436	1.06 (0.74–1.53)	0.747	1.04 (0.73–1.50)	0.818
	Quartile3	1.61 (1.19–2.18)	0.002	1.62 (1.16–2.26)	0.005	1.53 (1.09–2.15)	0.014
	Quartile4	1.67 (1.24–2.25)	0.001	1.64 (1.18–2.28)	0.003	1.33 (0.91–1.95)	0.141
		p for trend	0.001	p for trend	0.002	p for trend	0.047

Model 1 was a univariate analysis. Model 2 was adjusted for gender, age, duration of type 2 diabetes, body mass index, smoking habits, baseline concomitant disease, triglycerides, low-density lipoprotein cholesterol, blood pressure, anti-hyperglycemic therapy, and angiotensin-converting enzyme inhibitor or angiotensin II receptor blocker treatment. Model 3 was adjusted for the covariates of Model 2 plus average HbA1c.

HbA1c-adjSD, adjusted SD of HbA1c; HR, hazard ratio.

**Table 4:** Cox proportional hazards models of the HbA1c-CV with future clinical outcome.

Clinical outcome	HbA1c-CV	Model 1		Model 2		Model 3	
		HR (95%CI)	p	HR (95%CI)	p	HR (95%CI)	p
Renal events							
	Quartile1	Reference		Reference		Reference	
	Quartile2	1.42 (0.80–2.51)	0.228	1.40 (0.76–2.57)	0.286	1.37 (0.75–2.53)	0.309
	Quartile3	2.10 (1.22–3.62)	0.007	2.31 (1.28–4.14)	0.005	2.15 (1.19–3.90)	0.011
	Quartile4	2.58 (1.53–4.35)	<0.001	2.57 (1.46–4.52)	0.001	2.13 (1.15–3.94)	0.016
		p for trend	0.002	p for trend	0.003	p for trend	0.039
Diabetic eye disease events							
	Quartile1	Reference		Reference		Reference	
	Quartile2	0.70 (0.30–1.63)	0.410	0.64 (0.25–1.65)	0.358	0.63 (0.24–1.61)	0.329
	Quartile3	0.73 (0.31–1.73)	0.470	0.75 (0.30–1.86)	0.529	0.70 (0.28–1.76)	0.442
	Quartile4	1.43 (0.70–2.92)	0.333	1.24 (0.56–2.76)	0.600	1.02 (0.41–2.52)	0.965
		p for trend	0.268	p for trend	0.495	p for trend	0.653
Cardiovascular events							
	Quartile1	Reference		Reference		Reference	
	Quartile2	1.03 (0.60–1.76)	0.919	1.07 (0.59–1.96)	0.825	1.05 (0.57–1.92)	0.874
	Quartile3	0.88 (0.49–1.58)	0.658	0.68 (0.33–1.41)	0.291	0.62 (0.30–1.29)	0.198
	Quartile4	1.09 (0.63–1.89)	0.763	1.18 (0.64–2.18)	0.597	0.93 (0.47–1.84)	0.834
		p for trend	0.913	p for trend	0.487	p for trend	0.510
Cerebrovascular events							
	Quartile1	Reference		Reference		Reference	
	Quartile2	0.86 (0.42–1.79)	0.693	0.83 (0.37–1.89)	0.662	0.81 (0.36–1.84)	0.616
	Quartile3	1.69 (0.89–3.23)	0.109	1.38 (0.66–2.90)	0.395	1.27 (0.60–2.70)	0.530
	Quartile4	1.44 (0.74–2.80)	0.282	1.61 (0.78–3.34)	0.196	1.25 (0.57–2.78)	0.577
		p for trend	0.174	p for trend	0.301	p for trend	0.659
All-cause death							
	Quartile1	Reference		Reference		Reference	
	Quartile2	0.98 (0.54–1.76)	0.935	0.78 (0.40–1.53)	0.467	0.76 (0.39–1.50)	0.434
	Quartile3	1.57 (0.91–2.71)	0.108	1.87 (1.03–3.39)	0.039	1.72 (0.94–3.14)	0.080
	Quartile4	1.11 (0.62–2.01)	0.721	1.33 (0.71–2.49)	0.378	1.04 (0.51–2.09)	0.921
		p for trend	0.286	p for trend	0.039	p for trend	0.069
Composite endpoint events							
	Quartile1	Reference		Reference		Reference	
	Quartile2	1.14 (0.84–1.55)	0.405	1.05 (0.75–1.47)	0.768	1.03 (0.74–1.45)	0.846
	Quartile3	1.56 (1.16–2.09)	0.004	1.49 (1.07–2.06)	0.018	1.39 (1.00–1.94)	0.051
	Quartile4	1.52 (1.13–2.05)	0.006	1.53 (1.11–2.11)	0.010	1.27 (0.89–1.82)	0.180
		p for trend	0.006	p for trend	0.012	p for trend	0.164

Note. Model 1 was a univariate analysis. Model 2 was adjusted for gender, age, duration of type 2 diabetes, body mass index, smoking, baseline concomitant disease, triglycerides, low-density lipoprotein cholesterol, blood pressure, anti-hyperglycemic therapy, and angiotensin-converting enzyme inhibitor or angiotensin II receptor blocker treatment. Model 3 was adjusted for the covariates of Model 2 plus average HbA1c.

HbA1c-CV, the coefficient of variation of HbA1c; HR, hazard ratio.

## Discussion

This was the first study that focused on the effect of HbA1c trajectories on the future diabetes-related clinical outcomes in T2DM in China based on a 10-year follow-up cohort. HbA1c trajectories could reflect long-term HbA1c varying with time, which could be identified by several trajectory modeling techniques, such as LGMM, linear modeling, and latent group-based trajectory modeling (11, 15, 16). HbA1c trajectories groups referred to different clusters of individuals with an almost similar pattern of evolution of HbA1c and outcomes over time, and each subgroup must be large enough to ensure clinical relevance usually with a minimum of 1% of patients (11). Although HbA1c trajectories, HbA1c-adjSD, and HbA1c-CV could reflect long-term glucose fluctuation, the former is more easily understood due to intuitiveness. However, relative studies that involved HbA1c trajectories were limited. In our present study, three HbA1c trajectories were identified by LGMM, including low stable (88.34%), gradual decreasing (5.83%), and pre-stable and post-increase (5.83%). These findings suggested that most participants had well-controlled blood glucose, and several reasons could contribute to explaining this phenomenon. On the one hand, all the T2DM patients were regularly followed up by the community doctors to be given routine treatment and lifestyle guidance; on the other hand, specialists from Beijing Tongren hospital also made constructive suggestions for those with poor blood glucose control. Similarly, another study involved in HbA1c trajectories abroad also found that most participants were under good control of blood glucose in their cohort (17).

Although participants who had non-stable HbA1c trajectories also had higher HbA1c, and persistent hyperglycemia could directly affect renal function, Cox proportional HR models showed that participants who had non-stable HbA1c trajectories had a higher risk of renal events as compared to those who had the low stable pattern independent of average HbA1c, suggesting that the association between HbA1c trajectories and renal events did not depend absolutely on hyperglycemia. A Singapore study also demonstrated that participants with near-optimal stable trajectory had a lower risk of chronic kidney disease progression compared to those with moderate-stable or non-stable HbA1c trajectory independent of average HbA1c (9), but their sample size was smaller, and all the patients were from a diabetes center in a regional hospital. Nevertheless, our study further confirmed their findings with larger sample size and was more representative. Not only the pre-stable and post-increase but also the gradual decreasing trajectories had obviously blood glucose fluctuation compared to the low stable trajectory during the long-term glucose control progress, and glucose fluctuation was closely associated with diabetic complications. On the one hand, long-term glucose fluctuation could cause more oxidative stress and contribute to much severer endothelial dysfunction than hyperglycemia itself (7), which were important drivers of renal dysfunction. On the other hand, glucose fluctuation may cause renal injury *via* inducing inflammation damage and apoptosis injury in mouse glomerular mesangial cells (18). In addition, the gradual decreasing trajectory, started with high HbA1c but gradually decreased over time, had a higher risk of renal events, and this phenomenon may be partly due to the metabolic memory, namely, the risk of diabetic complications was strongly associated with previous glucose control (11). We also found the pre-stable and post-increase HbA1c trajectory had a higher risk of all-cause death and composite endpoint events. Despite the fact that more than 1/5 participants in the pre-stable and post-increase group began to use oral drugs combined with insulin to control blood glucose at baseline, they had HbA1c deterioration in the course of T2DM progress, indicating that the deterioration of HbA1c control is a potential risk factor for chronic comorbidities and death in T2DM patients. Our results indicated HbA1c trajectories may have additional effects on future clinical outcomes beyond average HbA1c, and low stable HbA1c trajectory may contribute to delaying complications in T2DM, so maintaining a sustained good glycemic control status was vital to preventing complications. Moreover, with the increasingly wide application of electronic medical records in clinical practice in China, it will be more available to plot a patient’s HbA1c trajectory, which may bring extra information for clinical decision-making and individualized treatment.

Although we found that the participants in the non-stable HbA1c trajectories groups had a higher risk of renal events, composite endpoint events, and all-cause death as compared to those in the low stable group, no significant association between HbA1c trajectories and macrovascular complications was shown. According to the United Kingdom Prospective Diabetes Study, the benefits of intensive glycemic control differed for the microvascular and macrovascular complications, which may be explained by time scale (11, 19–21). However, our follow-up period may be not long enough to discern different HbA1c trajectories’ effects on macrovascular complications, and therefore more longitude follow-up is needed. In addition, most participants had well-controlled blood glucose in our study, and participants with the clinical outcomes that occurred at stage I were also ruled out, which made the sample size of participants with macrovascular complications less.

Our study also found that higher HbA1c variability estimated by the HbA1c-adjSD and HbA1c-CV was associated with renal events and composite endpoint events in T2DM, which was in line with other studies (22, 23). However, the HbA1c-adjSD and HbA1c-CV could not intuitively reflect blood glucose change tendency, and its clinical value in prediction diabetic complications continued to be a debate due to summarizing complex, time-dependent phenomena into single measures (11, 24), while longitudinal HbA1c trajectories analyses can make up for this deficiency.

The present study has several strengths. First, our study introduced HbA1c trajectory as a newer indicator of glucose fluctuation to predict future clinical outcomes instead of exploring its association with other simple clinical indicators, and thus we provided more evidence for its values in clinical practice. Second, the present study covered 14 community service centers in 5 districts of Beijing, and the cohort was followed up for up to 10 years, making the follow-up data relatively complete and representative. Third, to better prove the predictive value of HbA1c trajectories, we divided the 10-year follow-up into two stages for the present data analysis to explore whether HbA1c trajectories could predict future clinical outcomes, while most studies could not clearly reflect the chronological order of them. The present study also had some limitations. First, the present study is not a random sampling survey based on a natural population, and the selection bias may exist to some extent. Second, it may take longer to observe the occurrence of macrovascular events, while the follow-up time was relatively short to observe the macrovascular events, and therefore more longitudinal studies are needed.

## Conclusion

Both HbA1c trajectories and HbA1c variability estimated by the HbA1c-CV and HbA1c-adjSD could predict future diabetes-related clinical outcomes. HbA1c trajectories could reflect long-term blood glucose fluctuation more intuitively. Non-stable HbA1c trajectories may predict increased risk of renal events, all-cause death, and composite endpoint events, independent of average HbA1c, and thus identifying HbA1c trajectories could be helpful for clinical decision-making.

## Data Availability Statement

The original contributions presented in the study are included in the article/supplementary material. Further inquiries can be directed to the corresponding author.

## Ethics Statement

The studies involving human participants were reviewed and approved by the Ethics Committee of Beijing Tongren Hospital. The patients/participants provided their written informed consent to participate in this study.

## Author Contributions

CM and WZ wrote the first draft of the manuscript and contributed to the collection and standardization of the data. GW conducted the data analysis and interpretation. RX, GY, XZ, HF, LZ, YJL, JZ, YL, YJ, DG, XC, ZW, and YC contributed to the discussion, conducted the research, and collected the data. SY contributed to designing the BCDS study.MY was the principal investigator, and designed this study and revised the manuscript. All authors read and approved the final manuscript.

## Funding

This project is supported by Capital’s Funds for Health Improvement and Research (CFH) (No.2011-2005-01, No.2016-1-2057, No.2022-1-1101), and BRIDGES Grant from the International Diabetes Federation (ST12-024).

## Conflict of Interest

The authors declare that the research was conducted in the absence of any commercial or financial relationships that could be construed as a potential conflict of interest.

## Publisher’s Note

All claims expressed in this article are solely those of the authors and do not necessarily represent those of their affiliated organizations, or those of the publisher, the editors and the reviewers. Any product that may be evaluated in this article, or claim that may be made by its manufacturer, is not guaranteed or endorsed by the publisher.

## Abbreviations

ACEI, angiotensin-converting enzyme inhibitors; ARB, angiotensin II receptor blockers; BMI, body mass index; DBP, diastolic blood pressure; FPG, fasting plasma glucose; HbA1c, hemoglobin A1c; HbA1c-CV, coefficient of variation of HbA1c; HbA1c-adjSD, adjusted SD of HbA1c; HbA1c-SD, SD of HbA1c; HDL-C, high-density lipoprotein cholesterol; HR, hazard ratio; IQR, interquartile range; LDL-C, low-density lipoprotein cholesterol; SBP, systolic blood pressure; T2DM, type 2 diabetes mellitus; TC, total cholesterol; TG, triglycerides; WC, waist circumference.
